# Forced Hepatic Expression of NRF2 or NQO1 Impedes Hepatocyte Lipid Accumulation in a Lipodystrophy Mouse Model

**DOI:** 10.3390/ijms241713345

**Published:** 2023-08-28

**Authors:** Nobunao Wakabayashi, Yoko Yagishita, Tanvi Joshi, Thomas W. Kensler

**Affiliations:** 1 Translational Research Program, Fred Hutchinson Cancer Center, Seattle, WA 98109, USA or yy3328@cumc.columbia.edu (Y.Y.); tjoshi@fredhutch.org (T.J.); tkensler@fredhutch.org (T.W.K.); 2 Division of Endocrinology, Columbia University, New York, NY 10032, USA

**Keywords:** NRF2, NQO1, KEAP1, lipodystrophy, lipogenesis, hydrodynamic tail vein injection, liver

## Abstract

Lipodystrophy is a disorder featuring loss of normal adipose tissue depots due to impaired production of normal adipocytes. It leads to a gain of fat deposition in ectopic tissues such as liver and skeletal muscle that results in steatosis, dyslipidemia, and insulin resistance. Previously, we established a *Rosa ^NIC/NIC^::AdiCre* lipodystrophy model mouse. The lipodystrophic phenotype that included hepatomegaly accompanied with hepatic damage due to higher lipid accumulation was attenuated substantially by amplified systemic NRF2 signaling in mice with hypomorphic expression of *Keap1*; whole-body *Nrf2* deletion abrogated this protection. To determine whether hepatic-specific NRF2 signaling would be sufficient for protection against hepatomegaly and fatty liver development, direct, powerful, transient expression of *Nrf2* or its target gene *Nqo1* was achieved by administration through hydrodynamic tail vein injection of *pCAG* expression vectors of dominant-active *Nrf2* and *Nqo1* in *Rosa ^NIC/NIC^::AdiCre* mice fed a 9% fat diet. Both vectors enabled protection from hepatic damage, with the *pCAG-Nqo1* vector being the more effective as seen with a ~50% decrease in hepatic triglyceride levels. Therefore, activating NRF2 signaling or direct elevation of NQO1 in the liver provides new possibilities to partially reduce steatosis that accompanies lipodystrophy.

## 1. Introduction

Lipodystrophy is a disorder that is attributed to a loss of selective functional adipose tissue due to absence of normal adipocytes. It leads to gain of fat deposition in ectopic tissues such as liver, skeletal muscle, and ovary in the case of females, accompanied by steatosis, dyslipidemia, and insulin resistance [1]. Previously, we established a lipodystrophy model mouse by constitutively promoting Notch signaling in adipose tissue specifically by generating *Rosa ^NIC/NIC^::AdiCre* mice [2]. The use of an *Adipoq Cre* recombinase construct fostered the dominant active expression of the Notch intracellular domain (NICD) beginning at postnatal day 4 in adipose tissues. Forced expression of Notch signaling engendered a loss of adipocytes in the adipose tissues in which lipogenesis and adipogenesis related gene expression was reduced. Consequently, lipids accumulated principally in the liver, thereby driving hepatomegaly and emergence of insulin resistance by 3 months of age.

Enhanced NF-E2-related factor 2 (NRF2) signaling through genetic manipulation of the pathway in these mice led to the prevention of hepatic steatosis, dyslipidemia, and insulin resistance by regulating hepatic lipogenic pathways and restoration of the hepatic fatty acid profile to the levels seen in control mice [3].

NRF2 is a transcription factor expressed ubiquitously in tissues. Its target genes were categorized initially as xenobiotic detoxication enzymes and oxidant/redox scavenging proteins (such as NAD(P)H quinone dehydrogenase 1 (NQO1); however, now a wide range of genes affecting cell fate and homeostasis have been defined that include anti-inflammatory, cell metabolism, and cell death pathways [4]. Within the cis-elements of target gene regulatory regions are antioxidant responsive elements (AREs) [5]. NRF2 associates with small MAF (musculoaponeurotic fibrosarcoma) proteins to bind to AREs to mediate positive or negative responses that can be cell-type dependent. Post-translational regulation of NRF2 is very important for maintaining cell homeostasis. NRF2 regulation is supported by ubiquitin-proteasomal degradation assisted by multiple molecular chaperones [6,7]. Kelch-like ECH associated protein 1 (KEAP1) is typically the most dominant NRF2-chaperone, and is localized principally in the cytosol to assemble with Cullin 3 (CUL3) and other accessory proteins into a NRF2 degron that leads to effective degradation of NRF2 [8,9]. The intensity of NRF2 target gene expression changes dramatically when *Keap1* is disrupted. These models include *Keap1* constitutive knockout mice, which exhibit postnatal lethality before weaning by malnutrition due to NRF2-driven esophageal constriction [10], heterozygotes that survive, as well as *Keap1* knockdown mice (*Kp1^A/A^*) [11] with constitutive hypomorphic *Keap1* expression without *Cre* transgene expression [12], or *Kp1^B/B^* [13] with wild-type level *Keap1* expression before tissue-specific *Cre* transgene expression.

Before the development of *Keap1*-disrupted mouse models, investigations on the roles of NRF2 in obesity and diabetes focused on exacerbation of disease in *Nrf2* knockout mice and protection by small molecule inducers of NRF2 signaling. The oleane triterpenoid CDDO-Im, which is known to target NRF2 signaling through binding to specific cysteine residues in KEAP1 [14], is partially preventive against high fat diet (HFD)-induced obesity. The inhibitory effect of CDDO-Im likely resulted from repression of de novo lipogenic genes such as *Fasn* and *Acc1*; reduction in the expression of these genes was abrogated in *Nrf2*-null mice [15]. This preventive effect against obesity, assumed to be mediated by enhanced NRF2 signaling by the inducer, was confirmed by the comparison between wild-type and *Kp1^A/A^* mice fed HFD. In this instance, NRF2 signaling repressed not only lipogenesis but also hepatic gluconeogenesis in mice on HFD [16].

An important feature of the lipodystrophy model was the observation that when the *Kp1^A/A^:: Rosa ^NIC/NIC^::AdiCre* mice had an additional deletion of the *Nrf2* gene, the beneficial physiology of the *Kp1^A/A^::Rosa ^NIC/NIC^::AdiCre* mice against lipodystrophy was lost, leading to diabetes [3]. In *Kp1^A/A^::Rosa ^NIC/NIC^::AdiCre* mice, white adipose tissues (WAT) did not recover; thus, enhanced NRF2 signaling did not affect normal WAT homeostasis in this model [3]. Therefore, in the current study, we generated *Kp1^B/B^:: Rosa ^NIC/NIC^::AdiCre* mice by breeding with *Rosa ^NIC/NIC^::AdiCre* and *Kp1^B/B^* mice to assess whether enhanced NRF2 signaling simply targeted to adipocytes could improve WAT mass, hepatic lipid accumulation, and tendency of liver damaging symptoms within the HFD setting. Given the incomplete protection in the *Kp1^B/B^:: Rosa ^NIC/NIC^::AdiCre* mice against the hepatic lipodystrophic phenotype in contrast to the *Kp1^A/A^:: Rosa ^NIC/NIC^::AdiCre* mice, we hypothesized that a key locus for enhanced NRF2 signaling might be in the liver. Therefore, hydrodynamic tail vein injection (HTI) [17] of a dominant active *Nrf2* (DA-*Nrf2*) expression vector into *Rosa ^NIC/NIC^::AdiCre* mice enabled enhanced NRF2 signaling with reasonable durability, directly in the liver. NQO1 is a classical NRF2 target gene. *Nqo1*-null mice exhibit significantly lower levels of abdominal adipose tissue mass along with higher hepatic levels of triglycerides in the adult [18], which is a similar phenotype of lipodystrophy. A *Nqo1* expression vector was also administered by HTI into *Rosa ^NIC/NIC^::AdiCre* mice. This strategy was highly protective against the development of fatty liver. Thus, in this report we introduce the protective effects of liver-specific enhanced NRF2 signaling as well as NQO1 into the lipodistrophy model *Rosa ^NIC/NIC^::AdiCre* mouse. Table 1 summarizes the lines of mice used for these studies.

## 2. Results

### 2.1. Characterization of Rosa^NIC/NIC^::AdiCre Mice

**Figure 1:**
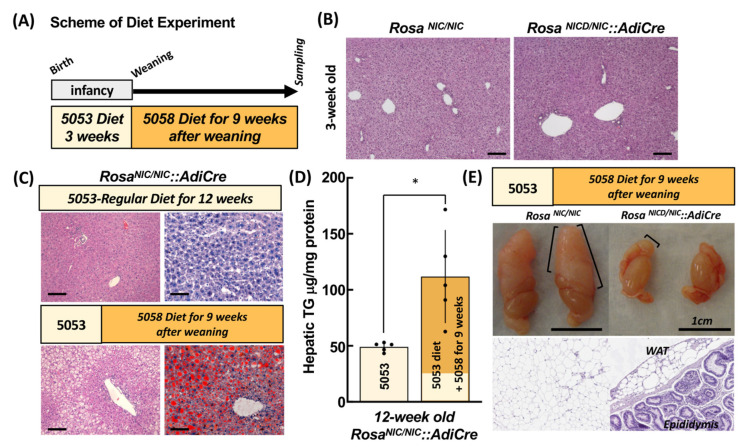
Lipodystrophy model in *Rosa^NIC/NIC^::AdiCre* mice. (**A**) Protocol for diet administration. Colors shown in (**A**) and used in (**C**–**E**) represent relative durations of 5053 and 5058 diets, respectively. (**B**) At 3 weeks of age, there was no difference between *Rosa^NIC/NIC^* mice and *Rosa^NIC/NIC^::AdiCre* mice in liver morphology (H and E). Scale bar: 100 µm. (**C**) Lipid accumulation phenotypes in the liver (H and E **left** and oil red-O staining **right**) of *Rosa^NIC/NIC^::AdiCre* mice after feeding nursing dams for 3 weeks with 5053 diet (4.55% fat) and switching to 5058 diet (9.0% fat) or continued maintenance with 5053 diet for an additional 9 weeks. Scale bar: 100 µm. Hepatic triglyceride (TG) levels in *Rosa^NIC/NIC^::AdiCre* mice are shown in (**D**). Data represent means ± SD of N = 5 mice. * *p* < 0.05 using *t*-test. (**E**) Atrophy of epididymal adipose tissue depicted as whole testes (**top**) and H and E staining of WAT (**bottom**) with switch to 9-week feeding with 5058 diet in *Rosa^NIC/NIC^::AdiCre* but not *Rosa^NIC/NIC^* mice.

Diet switches were used to induce lipodystrophy in this model (Figure 1A). Male *Rosa^NIC/NIC^::AdiCre* mice fed a regular 5053-diet containing 4.5% crude fat did not show hepatic lipid accumulation at 12 weeks of age with our current husbandry and housing conditions. We also could not see dramatic difference between *Rosa^NIC/NIC^* and *Rosa^NIC/NIC^::AdiCre* mouse liver at weaning before the experiments (Figure 1B). However, when the diet was switched to 5058-diet (PicoLab^®^ Rodent Diet 20 5058 containing 9% fat content) from 5053-diet at weaning (Figure 1A), higher amounts of hepatic lipids were observed (Figure 1C) at 12 weeks and triglyceride amount in the liver of *Rosa^NIC/NIC^::AdiCre* mice was increased by more than double of mice fed 5053-diet entirely (Figure 1D). Simultaneously, reduced WAT content was observed in the epididymal area (Figure 1E) as seen in the initial model [2].

### 2.2. Establishment of Kp1^A/A^::Rosa^NIC/NIC^::AdiCre and Kp1^B/B^::Rosa^NIC/NIC^::AdiCre Mice and the Comparison of Hepatic NRF2 Signaling 

**Figure 2:**
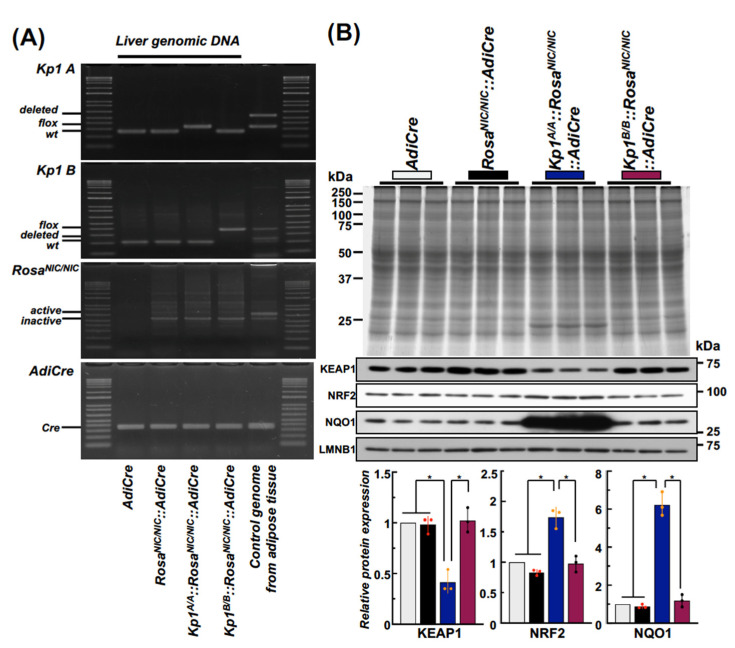
Differential NRF2 signaling between *Kp1^A/A^::Rosa^NIC/NIC^::AdiCre* and *Kp1^B/B^::Rosa^NIC/NIC^::AdiCre* compound mice. (**A**) Representative liver genomic DNA confirmation for lack of off-target effects by *AdiCre* in the mouse lines used. (**B**). The distinctive hepatic expression of NRF2 signaling (NQO1 levels) of the hypomorphic *Kp1^A/A^::Rosa^NIC/NIC^::AdiCre* mice was confirmed by immunoblot analyses (N = 3 for each genotyped mouse). Mercury, black, blue, and maroon boxes show whole liver protein samples and KEAP1, NRF2 and NQO1 results from *AdiCre*, *Rosa^NIC/NIC^::AdiCre, Kp1^A/A^ ::Rosa^NIC/NIC^::AdiCre* and *Kp1^B/B^::Rosa^NIC/NIC^::AdiCre* compound mice, respectively. LMNB1 expression was used for normalizing for the quantification of each protein blot shown in the histograms. * *p* < 0.05 using one-way ANOVA followed by Tukey’s test (highlighted by red data points).

None of the mouse lines showed the deletion/activation of the floxed allele by unexpected *Cre* expression in the liver genomic DNA. For genomic DNA isolated from adipose tissue, *Keap1* deleted allele or *Rosa^NIC/NIC^* expressing allele bands were detected at ~450 bp (*Kp1^A/A^* deleted), ~380 bp (*Kp1^B/B^* deleted), and ~650 bp (NICD expression). The *Cre* gene functioned in adipose tissue (Figure 2A) reflecting the specificity of *Adipoq* gene expression previously reported [3]. In the liver isolated from 12-week-old male mice of each line fed with 5053-diet, NRF2 signaling related genes products were analyzed by immuno-blotting (Figure 2B). *Kp1^A/A^::Rosa^NIC/NIC^::AdiCre* mice with dampened (hypomorphic) *Keap1* gene expression exhibited the highest hepatic NRF2 amount together with elevation of its representative gene target NQO1 among all the genotypes. Furthermore, a similar sized band at ~22 kDa, likely a μ or π class GST, was observed in the liver of *Keap1* hypomorphic mice, (Figure 2B CBB-staining) [10]. By contrast, *Kp1^B/B^::Rosa^NIC/NIC^::AdiCre* mice exhibited similar levels of KEAP, NRF2 and target gene products as the control mice. These results indicate that comparing NRF2 signaling on the fatty liver phenotype between *Kp1^A/A^::Rosa^NIC/NIC^::AdiCre* and *Kp1^B/B^::Rosa^NIC/NIC^::AdiCre* mice could allow assessments of the role of hepatic as opposed to adipocyte NRF2 on the hepatic symptoms of lipodystrophy in this model.

### 2.3. Differential Hepatic Lipid Accumulation among the Genetic Mutant Mice

**Figure 3:**
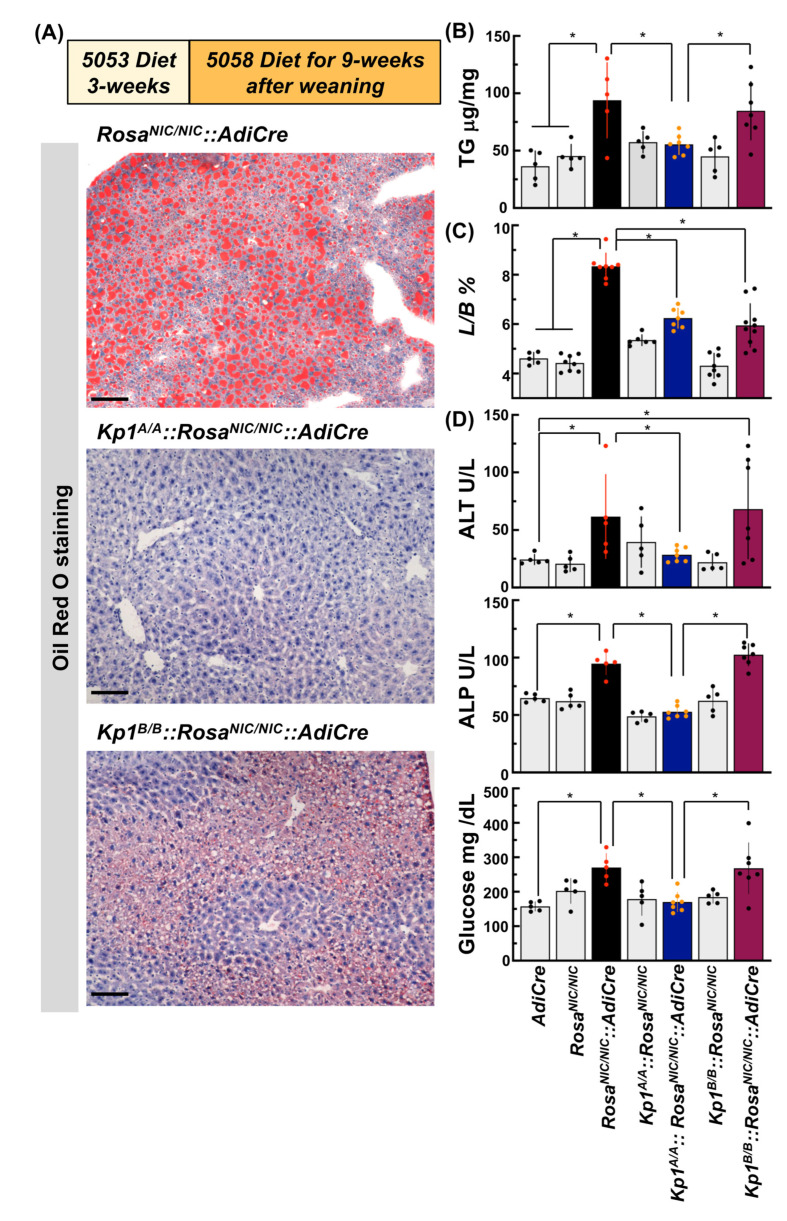
Effect of two *Keap1* genotypes in the lipodystrophy model. (**A**) Hepatic oil red-O staining of *Rosa^NIC/NIC^::AdiCre*, hypomorphic (*Kp1^A/A^*) and normal floxed (*Kp1^B/B^*) genotypes. Scale bar: 100 µm. (**B**) Hepatic TG quantification following the diet switch experiments. Data were analyzed as per mg protein in each mouse liver extract and represent means ± SD of N ≥ 5 mice. * *p* < 0.05 using ANOVA followed by Tukey’s test. (**C**) Effects of hypomorphic (*Kp1^A/A^*) and normal floxed (*Kp1^B/B^*) genotypes on percent of liver weight per whole body weight (L/B%); (**D**) serum ALT, ALP activities, and ad libitum-fed blood glucose levels. * *p* < 0.05 using ANOVA followed by Tukey’s test or Dunnett’s multiple comparison test (ALT) (N ≥ 5) (highlighted by red data points).

Following the diet protocol with a switch to a higher fat diet at 3 weeks as indicated in Figure 1A, *Rosa^NIC/NIC^::AdiCre* mice invariably showed hepatomegaly and severe ectopic lipid accumulation in the liver at 12 weeks (Figure 3A–C). *Kp1^A/A^::Rosa^NIC/NIC^::AdiCre* mice exhibited much milder lipid accumulation than *RosaNIC/NIC::AdiCre mice* where hepatic triglyceride levels were comparable to those of control mice (Figure 3B). This effect in the liver has been considered as a reflection of amplified NRF2 signaling by both ubiquitous hypomorphic *Keap1* expression, which is specific characteristic of *Kp1^A/A^* background mice, in combination with complete *Keap1* deletion in the adipose tissues due to *Cre* expression based on the control of the *Adipoq* promoter.

Since there are no reports of knockdown of *Keap1* expression in the tissues of *Kp1^B/B^* mice, this line was examined to determine whether adipose tissue specific *Keap1* deletion could contribute to a milder liver phenotype than observed in *Rosa^NIC/NIC^::AdiCre* mice. As shown in Figure 3C, *Kp1^B/B^::Rosa^NIC/NIC^::AdiCre* mice seemed to be restrained in manifesting the phenotype of enlarged liver size seen in *Rosa^NIC/NIC^::AdiCre* mice, a protection also observed in the *Kp1^A/A^::Rosa^NIC/NIC^::AdiCre* mice. However, *Kp1^B/B^::Rosa^NIC/NIC^::AdiCre* mice, unlike *Kp1^A/A^::Rosa^NIC/NIC^::AdiCre* mice, did not prevent hepatic lipid accumulation and increased levels of hepatic triglycerides, ALT, AST, and serum glucose that were observed in *Rosa^NIC/NIC^::AdiCre* mice (Figure 3A–C).

### 2.4. The Application of DNA-HTI into Mice

**Figure 4:**
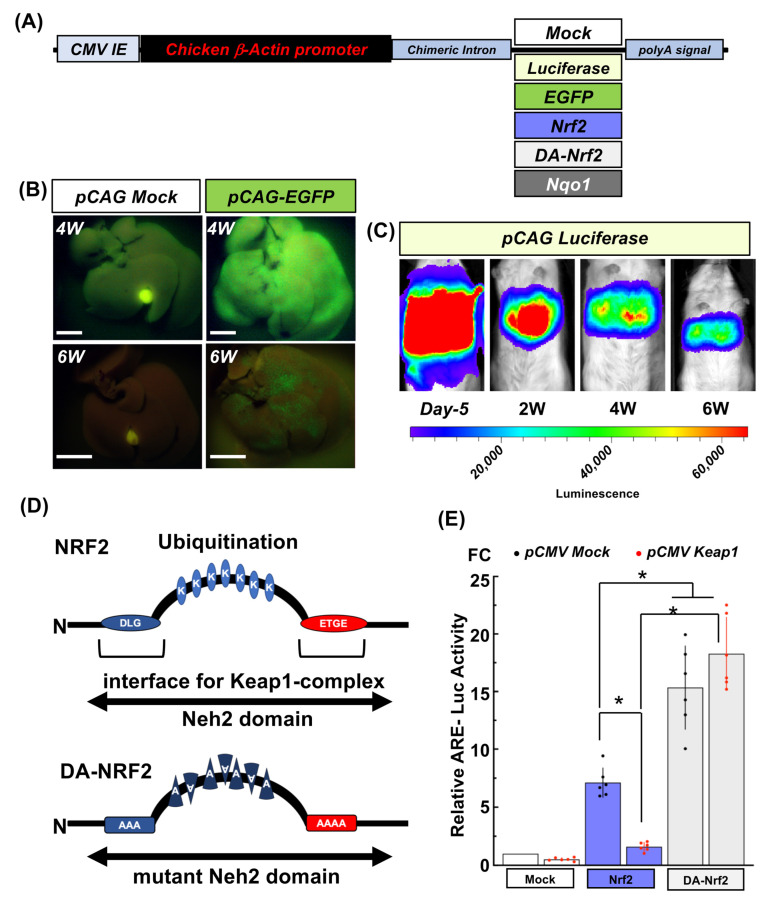
Confirmation of hepatic gene expression dynamics following hydrodynamic tail vein injections (HTI) of *pCAG* reporter genes and effect of DA-*Nrf2* to enhance NRF2 and target gene expression in MEF. Each vector is shown in (**A**). Time course for EGFP or luciferase reporter gene expression in the livers of C57BL6/J albino mice after injection with *pCAG EGFP* (for 4W *pCAG Mock* shutter speed at 1 s and 4 W *pCAG-EGFP* at 1/8 s with an Olympus IF550 filter) (Scale bar: 5 mm), (**B**) or *pCAG Luc*, (**C**) by HTI. (**D**) Differences in Neh2 domains between NRF2 and sites of mutation in dominant active-NRF2 (DA-*Nrf2*). (**E**) Functional differences between NRF2 and DA-NRF2 on ARE-reporter gene expression in wild type MEF transfected with *pCAG Mock*, *pCAG Nrf2*, or *pCAG* DA*-Nrf2*. Red dots or black dots indicate luciferase activities from MEFs co-transfected with *pCMV Keap1* or its *pCMV Mock*, respectively. * *p* < 0.05 using ANOVA followed by Tukey’s test.

To provide powerful and sustainable gene expression in the liver, *pCAG* was selected as the expression vector [19,20] for this purpose (Figure 4A). Both *pCAG EGFP* and *pCAG Luciferase* reporter genes were tested to determine how long the p*CAG* vector would continue to express the gene following HTI in 5-week-old male mice. In all trials, *pCAG EGFP* gene expression was still detected at 6 weeks after HTI, although its expression began to quickly diminish around 4 weeks after HTI (Figure 4B). In vivo luciferase activity derived from the *pCAG Luciferase* vector was traced from 5 days to 6 weeks after HTI with an in vivo imaging system (IVIS). Luciferase activity was detected at each time point but exhibited a similar tendency to diminish expression at 4–6 weeks after HTI (Figure 4C). Nonetheless, luciferase detection by IVIS remained positive until 10 weeks after HTI. Through in vivo reporter gene analyses, we confirmed that the *pCAG* vector could drive persistent gene expression in the liver following delivery by HTI.

### 2.5. Development of a Dominant Active-Nrf2 Expression Vector

To produce a vector for sustainable NRF2 signaling in mouse liver, we prepared a dominant active type of recombinant NRF2 (DA-*Nrf2*) expression vector. The Neh2 domain in NRF2 is critical for facilitating its degradation by the KEAP1-degrasome coupled with CUL3. The negative regulation of NRF2 is maintained through its DLG and ETGE subdomains within Neh2 to form the interface with KEAP1. Seven lysines (Ks), which are positioned between the DLG and ETGE subdomains, are sites for ubiquitination and marking for proteasomal degradation [21,22,23,24]. Therefore, these critical amino residues were altered into alanine in DA-*Nrf2*, which produces much less affinity to KEAP1 and avoids quick degradation (Figure 4D). As shown in MEFs in Figure 4E, the activity of an ARE reporter by DA-*Nrf2* was 2-fold higher than with wild-type NRF2. Moreover, ARE reporter activity did not decrease despite co-transfection of a *Keap1* expression vector into the cells, unlike in the case of transfection of a wild-type *Nrf2* expression vector.

### 2.6. Forced Expression of Nrf2 and Its Target Gene Nqo1 in Liver by HTI and Its Effects One Week Later on NRF2 Target Gene Expression

**Figure 5:**
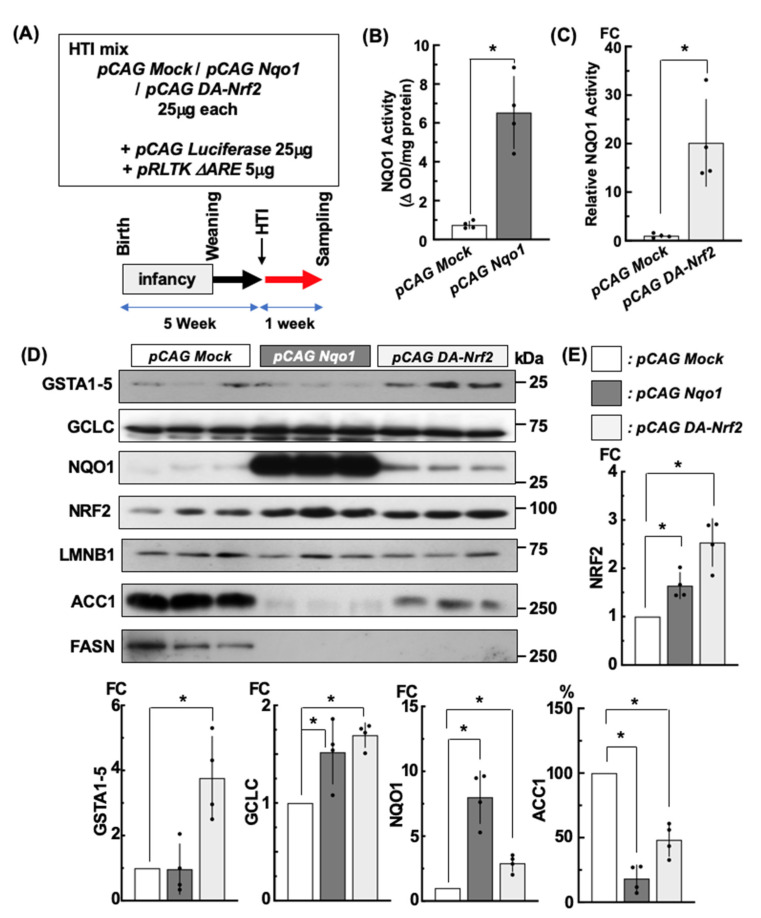
Effect of DA-NRF2 and NQO1 expression in hepatic NRF2 signaling by HTI of *pCAG Nqo1* or *pCAG* DA*-Nrf2* in mouse. (**A**) HTI DNA cocktail and experimental scheme is depicted. Samples were harvested one week after HTI from 5-week-old male mice. NQO1 activity is shown in (**B**,**C**). (**B**) is normalized by total protein and (**C**) by the Renilla luciferase activity derived from the internal control vector *pRLTKΔARE* included in the HTI mixture. (**D**) Immunoblotting analyses of NRF2 signaling gene products using whole liver protein lysate isolated one week after HTI with each *pCAG* vector. * *p* < 0.05 using ANOVA followed by Tukey’s test in (**E**) and using *t-*test in (**B**,**C**). N = 4; individual data points indicated.

The expression of *pCAG Nqo1* and *pCAG* DA*-Nrf2* was confirmed in vivo at 1 week after HTI (Figure 5A). Each vector product was detected at a higher level than the control vector in each liver extract. Additionally, immunohistochemical staining of the liver of *pCAG Nqo1* treated mice revealed increased expression of NQO1 in hepatocytes, principally around the central vein (Supplementary Figure S4). *pCAG* DA*-Nrf2* affected the canonical expression of direct NRF2 target genes including *Nqo1*, *Gsta*s, and *Gclc*. Interestingly, the liver extract isolated from *pCAG Nqo1* HTI showed higher levels of endogenous *Nrf2* expression along with *Gclc,* but not *Gsta*s (Figure 5D,E). The *pCAG Nqo1* driven NQO1 activity was 6.5 times fold higher than in the control *pCAG Mock* HTI liver samples. The injected *pCAG* DA*-Nrf2* dependent NQO1 activity normalized with Renilla luciferase activity derived from co-injected *pRLTKΔARE* was also at a higher level than with the *pCAG Mock vector* (Figure 5B,C). To examine the influence on de novo lipogenesis in the liver following HTI of each vector, *Acc1* and *Fasn,* which are considered as negatively regulated NRF2 target genes [3,15,25], were analyzed by immunoblotting analyses. Remarkably, both ACC1 and FASN levels were greatly reduced in the livers treated with *pCAG Nqo1* or *pCAG* DA*-Nrf2* HTI (Figure 5D,E). Taken together, this experimental approach presaged an expectation that enhancing NRF2 signaling by HTI might prevent the hepatic symptoms of lipodystrophy in the *Rosa^NIC/NIC^::AdiCre* mice.

### 2.7. Forced Hepatic Expression of Nrf2 and Its Target Gene Nqo1 of Rosa^NIC/NIC^::AdiCre Lipodystrophy Model Mice

**Figure 6:**
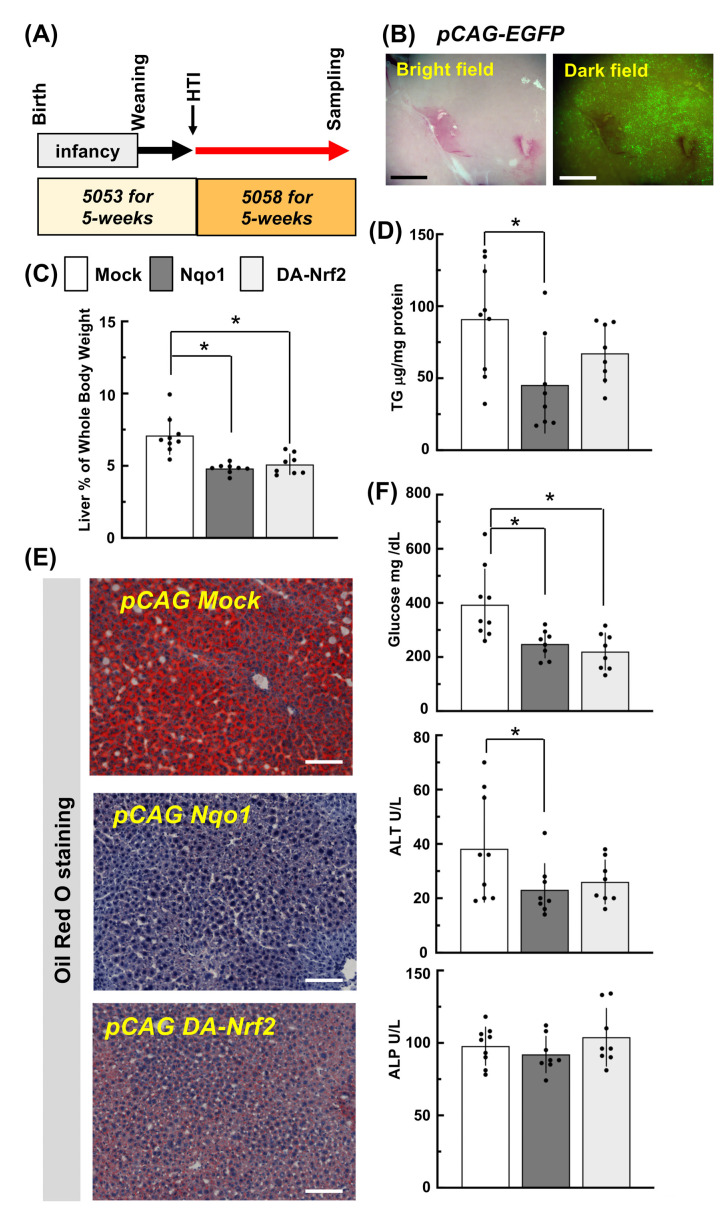
Protection from hepatic lipid accumulation in *Rosa^NIC/NIC^::AdiCre* mice by HTI of DA-*Nrf2* or *Nqo1* expression vectors. (**A**) Experimental scheme for diets and HTI timing. (**B**) Representative EGFP expression in the liver at 5 weeks after HTI. Livers were perfused with cold Hank’s buffer to remove blood before observation. Scale bar: 500 µm. (**C**) Percent of liver weight per whole body weight after HTI of each expression vector and 5-weeks feeding of 5058 diet. (**D**) Hepatic triglyceride amounts. Data represent means ± SD of N ≥ 7 mice (individual data points indicated). * *p* < 0.05 using by ANOVA followed Dunnett’s multiple comparison test. (**E**) Representative oil red-O staining in liver sections following HTI of each vector and diet switch. Scale bar: 100 µm. (**F**) Serum ALT, ALP activities and ad libitum-fed blood glucose levels. * *p* < 0.05 by ANOVA followed by Tukey’s test or unprotected Fisher’s test (ALT).

Shown in Figure 6A, each of the *pCAG Mock*, *pCAG* DA*-Nrf2*, or *pCAG Nqo1* vectors were injected by HTI method into 5-week-old male *Rosa^NIC/NIC^::AdiCre* mice. Immediately after the injection, the 5053-diet was switched to the higher fat 5058-diet for the following 5 weeks. Under this dietary protocol, the liver sections prepared from *Rosa^NIC/NIC^::AdiCre* mice injected with *pCAG Mock* constantly exhibited oil red O positive hepatic cells (Figure 6E) at the time of liver harvest as expected. A similar EGFP expression pattern as seen previously (Figure 4) in mice was observed in these mice co-injected with *pCAG EGFP* (Figure 6B). This result led to an expectation that NQO1 or DA-NRF2 derived from each *pCAG* vector would be expressed to at least similar level as EGFP in the livers. Importantly, the hepatomegaly seen in *Rosa^NIC/NIC^::AdiCre* mice injected with *pCAG Mock* was attenuated in mice injected with p*CAG Nqo1* or *pCAG* DA*-Nrf2* (Figure 6C). Further, hepatic triglyceride levels were decreased by 50% in mice injected with *pCAG Nqo1* compared to *Rosa^NIC/NIC^::AdiCre* mice injected with *pCAG Mock,* but less so by *pCAG* DA*-Nrf2*. Liver sections stained by oil red O also reflected the results of hepatic triglyceride levels (Figure 6D,E). Except for plasma ALP, blood glucose, and ALT levels in *Rosa^NIC/NIC^::AdiCre* mice injected *pCAG Nqo1*or *pCAG* DA*-Nrf2* were reduced by nearly 50% compared to the mice injected *pCAG Mock* vector. Thus, HTI administration of *Nrf2* and *Nqo1* expression vectors provided dramatic protection against aspects of hepatomegaly and lipid accumulation in this model.

## 3. Discussion

Lipodystrophy syndromes usually present with several metabolic abnormalities associated with insulin resistance that include diabetes mellitus, hypertriglyceridemia, and hepatic steatosis [26]. Pharmacologic or systemic genetic activation of the NRF2 pathway (e.g., *Kp1^A/A^* hypomorphic) partially protects mice fed a high-fat diet (60% kcal fat versus 10%) from obesity and insulin resistance [15,16]. Key lipogenic enzymes were repressed in the livers of the *Keap1* hypomorphic mice as were triglyceride levels. In the same model, cell-specific deletion of *Nrf2* from adipocytes but not hepatocytes potentiated systemic metabolic dysfunction. Hepatocyte-specific *Nrf2*-disrupted mice showed no difference in hepatic triglyceride accumulation compared to wild-type mice fed the high-fat diet [27]. A second model was developed, as used in the current study, in which C57Bl/6J *Rosa ^NIC/NIC^::AdiCre* mice were generated to overexpress NICD specifically in adipocytes. These mice developed a phenotype of lower body fat mass and higher serum levels of glucose, insulin, and triglycerides leading to severe insulin resistance and steatotic livers [2]. This phenotype was verified in the feeding regimen we employed in this study.

The goal of the present study was to compare the influence of systemic (*Kp1^A/A^*) amplification of NRF2 signaling to that of adipocyte-specific amplification in *Kp1^B/B^* mice on fatty liver disease. *Kp1^B/B^* mice had been developed to probe the role of enhanced expression of NRF2 signaling in tissue-specific manners in the lung on cigarette smoke-induced oxidative stress and inflammation [13], and subsequently in T-cells, myeloid cells, and dendritic cells in autoimmune inflammation [28], myeloid leukocytes in sepsis [29], and kidney epithelium in hydronephrosis [30]. There are no reports of off-target hypomorphism of *Keap1* expression in these mice. Tamoxifen-inducible, whole body *Cre*-mediated deletion of *Keap1* in the *Kp1^B/B^* line improved glucose homeostasis and insulin sensitivity compared to *Kp1^B/B^* controls [31]. While the phenotypes of hepatic triglyceride accumulation and hepatomegaly in *Rosa ^NIC/NIC^::AdiCre* mice were reduced considerably in *Kp1^A/A^::Rosa^NIC/NIC^::AdiCre* mice; concomitant deletion of *Nrf2* from these mice nullified the hepatic phenotype. These observations indicate that increased hepatic NRF2 signaling might contribute to the prevention of lipodystrophic symptoms. Therefore, the comparison of the *Kp1^A/A^::Rosa^NIC/NIC^::AdiCre* and *Rosa ^NIC/NIC^::AdiCre* mouse combined into the *Kp1^B/B^* background was an important first step for testing our hypothesis. Adipose tissue-specific *Keap1* deletion did not reduce hepatic triglyceride accumulation to a statistically significant degree relative to *Kp1^A/A^::Rosa^NIC/NIC^::AdiCre* mice even though the hepatomegaly phenotype was largely mitigated in both *Kp1* floxed lines. Protein levels of KEAP1 were reduced, and NRF2 levels along with its canonical target NQO1 were upregulated in livers of *Kp1^A/A^* but not *Kp1^B/B^* mice. Constitutive *Keap1* hypomorphic expression contributed to prevent the lipid accumulation and evade its lipotoxicity in the liver as measured through ALT activity. Perhaps the mitigation of fatty liver in this model reflects both diminished lipid mobilization from adipocytes to ectopic sites as well as enhanced hepatic lipid metabolism driven by hepatic (and possibly extra-hepatic) amplification of NRF2 signaling. Indeed, blood glucose levels were decreased in *Kp1^A/A^::Rosa^NIC/NIC^::AdiCre* mice but not in *Kp1^B/B^* background mice, suggesting systemic metabolic effects driven by *Keap1* hypomorphic expression are one of the key elements in inter-organ communication that is disturbed in the setting of lipodystrophy. In this study, we narrowed down our focus on the role of NRF2 signaling to the liver, a hub metabolic organ, in *Rosa ^NIC/NIC^::AdiCre* mice.

The same sequences for *lox P* elements lie on both *Rosa ^NIC/NIC^::AdiCre* and *Kp1^B/B^* alleles to activate *NICD* or delete *Keap1,* respectively. Thus, the *Rosa*-*Keap1* mutant mice employing a *Cre* expression vector precludes upregulating NRF2 signaling specifically in the liver. To provide a direct evaluation of hepatic NRF2 signaling in mitigating the lipodystrophic phenotype of the *Rosa ^NIC/NIC^::AdiCre* mice, a *pCAG*-driven dominant-active *Nrf2* expression vector was directed to the liver by HTI [32]. The use of the DA-*Nrf2* expression vector, designed to impede NRF2 degradation, provides a persistent hepatic expression of NRF2 over a period of weeks. HTI of the *pCAG DA-Nrf2* expression vector into *Rosa ^NIC/NIC^::AdiCre* mice reduced hepatomegaly, serum ALT activity, and glucose levels, but only partially diminished hepatic triglyceride levels or oil red O staining. However, hepatic levels of NRF2 were increased nearly 3-fold compared to *pCAG Mock* after 1 week and downstream gene product proteins were elevated 4-fold, 3-fold, and 2-fold for GSTA, NQO1, and GCLC, respectively. A robust hepatic NRF2 response was evoked.

Impressively, HTI of a *pCAG Nqo1* expression vector afforded dramatic reductions in oil red O staining and a 50% drop in hepatic triglyceride levels. Complementing these outcomes, this expression vector elevated hepatic NQO1 protein levels 7-fold and provoked a modest but statistically significant 50% increase in NRF2 levels. Indeed, in the case of *pCAG-Nqo1* HTI liver, NQO1 could be detected at 9 weeks following HTI of *pCAG Nqo1* into *Rosa ^NIC/NIC^::AdiCre* mice by immunohistochemically (Supplementary Figure S4). This strong protective effect of elevated hepatic NQO1 was presaged by earlier reports in the literature in studies employing *Nqo1*-knockout and *Nqo1*-transgenic mice [31].

Lipodystrophy is best characterized by the movement of fat from adipose tissue to the liver. Yet, based on previously published information [3] along with the results of the current study, the mitigation of lipid accumulation in the liver of *Rosa^NIC/NIC^::AdiCre* mice might be caused partially by the decreased expression of hepatic de novo lipogenesis related genes. Forced expression of NRF2 derived from *pCAG DA-Nrf2* may upregulate endogenous *Nrf2* that bears a functional ARE on its promoter [33]. NRF2 increase was also observed in the *pCAG Nqo1* HTI liver. There might be direct downregulation of *Acc1* and *Fasn* genes. Indeed, putative functional ARE sequences are observed in the regulatory regions of both genes proximal from the transcriptional initiation site. It is reported that well-established lipodystrophy mouse models, such as *Agpat2* disrupted mice and *A-ZIP/F* transgenic mice, develop severe lipodystrophy along with an elevation of a hepatic pool of triglycerides and upregulated hepatic de novo lipogenesis [34,35], suggesting that steatosis observed in lipodystrophy is complex, and hepatic de novo lipogenesis is, at least partially, one of the metabolic factors that contribute to steatosis. Interestingly, Cortes et al. demonstrated leptin replacement in *Agpat2*^−/−^ mice ameliorated hepatic function, wherein improvement was partially driven by a decrease in hepatic de novo lipogenesis [36]. Thus, we consider that suppressed expression of lipogenesis-related enzymes (i.e., ACC1 and FASN) in the livers of *pCAG* DA*-Nrf2* and *pCAG Nqo1* treated *Rosa^NIC/NIC^::AdiCre* mice is a part of the multifactorial mechanisms underlying improvement of steatosis in the setting of lipodystrophy.

NQO1 has multiple roles in the control of redox processes relevant to several disease states including metabolic syndrome. Its roles as a molecular chaperone [37] and an inhibitory function of proteasomal degradation systems [38] have expanded recently. It is well-known that NRF2 is regulated by the ubiquitin proteasomal system [7,8,9] given our observation of increased NRF2 levels in the *pCAG Nqo1* HTI treated liver, NQO1 might be able to protect NRF2 from proteasomal degradation and amplify its signaling capacity. The possible NQO1 function of the linkage to lipodystrophic related genes products and its mechanism should be elucidated in *Rosa^NIC/NIC^::AdiCre* mice.

In humans, the NQO1*2 null polymorphism has been associated with increased risk of adverse lipid profiles and Type 2 diabetes [39,40]. Gaikwad et al. [18] utilized *Nqo1*-knockout mice to examine the role of this enzyme on intracellular redox states and lipid metabolism and distribution. Substantial reductions in the amount of abdominal adipose tissue and elevated hepatic triglyceride levels were reported, not unlike the alterations observed in the *Rosa^NIC/NIC^::AdiCre* mice. Pharmacological activation of NADH oxidation by NQO1 through administration of the substrate β-lapachone has been associated with amelioration of obesity along with glucose intolerance, dyslipidemia, and fatty liver [41,42]. More directly, Di Francesco et al. [31] demonstrated that NQO1-transgenic mice on a high fat diet exhibited enhanced expression of lipogenic enzymes coincident with reductions in circulating and hepatic lipids.

Systemic gain of NRF2 signaling produces ameliorative effects against the ectopic lipogenic phenotype in livers of *Rosa^NIC/NIC^::AdiCre* mice [3,15,16,27]. Liver specific gain of NRF2 signaling following HTI of *pCAG* DA*-Nrf2* or more effectively *pCAG Nqo1* vectors also lead to effective mitigation of damage to the liver. NQO1 might be the practical NRF2 target gene for reduction in hepatic lipodystrophic symptoms through interrupting de novo lipogenesis with positive feedback of NRF2 signaling. If transient forced expression of *Nqo1* could enhance NRF2 signaling, NQO1 itself may be the critical mediator for preventing lipodystrophic symptoms like fatty liver. As a pharmacological target, screening for the most potent and effective in vivo inducers of hepatic NQO1 might be fruitful. Selective elevation of or mimetics of NQO1 chaperone function might be useful but will require elucidation of the functional domains of NQO1 as a molecular chaperone.

## 4. Materials and Methods

### 4.1. Animals

Mice were maintained at 22 °C, 50% humidity with a 12-h-light/dark cycle with ad libitum access to water and food (PicoLab^®^ Rodent Diet 20 5053 (or 5058 as when noted) irradiated diet, LabDiet, Arden Hills, MN, USA). All mice were the albino C57BL/6J background (B6(Cg)-Tyrc-2J/J) (Jackson Laboratories, Bar Harbor, ME, USA). *Keap1* floxed mice, *Kp1^A/A^* [11] and *Kp1^B/B^* [13] were obtained from Prof. M. Yamamoto, Division of Medical Biochemistry, Tohoku University School of Medicine, Sendai, Japan, and Prof. S. Biswal, Johns Hopkins Bloomberg School of Public Health, Baltimore, MD, USA, respectively. B6;FVB-Tg(Adipoq-cre)1Evdr/J [43] (*AdiCre*) and Gt(Rosa)26Sortm1(Notch1)Dam/J [44] (*Rosa^NIC/NIC^*) were purchased from Jackson Laboratories (Bar Harbor, ME, USA). *Kp1^A/A^* or *Kp1^B/B^::Rosa^NIC/NIC^::AdiCre* mice were generated by crossing *Kp1^A/A^* or *Kp1^B/B^::Rosa^NIC/NIC^::AdiCre* with *Kp1^A/A^* or *Kp1^B/B^::Rosa^NIC/NIC^*, which were established by consecutive mating of *Kp1^A/+^* or *Kp1^B/+^::Rosa^NIC/+^::AdiCre* with *Kp1^A/+^ or Kp1^B/+^::Rosa^NIC/+^* mice. In this study, male *AdiCre* carrier mice were used for maintaining the mouse line. All mice were evaluated by standard PCR-genotyping. The PCR conditions and primers, and locations used for genotyping of each line are described in Supplementary Figures S1 and S2, Tables S1 and S2, and previous work [3]. The mouse genotypes used in this research are listed in Table 1. For the regular diet experiment, 3-week-old (8–12 g) male mice N ≥ 5/genotype were used for the protocol shown in Figure 1A. In the case of diet experiment with HTI, 5-week-old (20–25 g) male mice N ≥ 4 for wild-type mice or N ≥ 5 for *Rosa^NIC/NIC^::AdiCre* mice were utilized. All mouse experiments were performed at the FHCC and were approved by the Institutional Animal Care and Use Committee (protocol #51042).

### 4.2. Blood Collection for Biochemical Analyses and Dissection of Liver

Mice were anesthetized using an isoflurane (Piramal Critical Care, Bethlehem, PA, USA) vaporizer (Surgivet model 100, Smiths Medical North America, Waukesha, WI, USA). Continuously under isoflurane anesthesia, mice were cut with a Y-shaped incision along the abdominal surface using sterilized surgical tools; intestines were gently moved to right side. Blood was collected through the inferior vena cava using heparinized 25 G needle with 1 mL syringe [45]. Blood was kept on ice until centrifugation at 3000× *g* for 30 min at 4 °C for plasma isolation. The plasma biochemical analyses were conducted by commercial laboratories (Zoetis Reference Laboratories, Mukilteo, WA, USA, or Moichor Reference Labs, San Francisco, CA, USA). Following blood collection, the entire liver was dissected and weighed. One third of both the center and left lobes were cut for the histological analyses, the remaining liver was flash frozen and kept at −80 °C prior to molecular and biochemical analyses. Mice were euthanized by cervical dislocation and 1 mm of each tail was cut and genomic DNA isolated for confirmation of genotype.

### 4.3. Triglyceride Assay

Approximately 100 mg of liver was used for assay with the Cayman 10010303 triglycerides assay kit (Cayman, Ann Arbor, MI, USA). Spectrophotometric measurement was performed using a SpectraMax M5 plate reader (Molecular Devices, San Jose, CA, USA). Protein concentration was measured using the BCA reagent (Thermo Fisher Scientific, Waltham, MA, USA), which was used for the normalization of enzyme activity.

### 4.4. NQO1 Enzyme Assay 

Approximately 100 mg of liver was lysed with 0.08% digitonin/EDTA (2 mM, pH 7.8) for 20 min at 37 °C. The assay was performed as described previously [46]. Briefly, 80 μL of cell lysate was incubated at room temperature for 5 min with 200 μL of the reaction mixture: NQO1 assay buffer (25 mM Tris, pH 7.4, 0.66 mg/mL bovine serum albumin, 0.01% Tween-20) mixed with cofactors (5 mM flavin adenine dinucleotide, 1 mM G6P, 30 mM NADP, 30 U G6P, 0.3 mg/mL MTT) and 50 mM menadione. Spectrophotometric measurement was performed using SpectraMax M5 plate reader (Molecular Devices). BCA reagent (Thermo Fisher Scientific, Waltham, MA, USA) was used to measure protein concentration for normalizing enzyme activity.

### 4.5. In Vivo Imaging and Stereo-Microscopic Observation of Livers Targeted with Luciferase or EGFP Reporter Genes by Hydrodynamic Tail Vein Injection 

All DNA was isolated and purified with endotoxin-free Qiagen Maxi prep (Qiagen, Germantown, MD, USA). For hydrodynamic tail vein injections, TransIT-EE Hydrodynamic Delivery Solution (Mirus, Madison, WI, USA) was utilized. Then, 25 μg of *pCAG Luciferase* or *pCAG EGFP* was injected with the delivery solution [formulated as 1/10 of mouse body weight (g) as mL + void volume 100 μL (including DNA solution)] into male 5-week-old albino C57BL/6J mice to examine the time-course of reporter gene expression. Sterilized (0.2 μm-filter) D-luciferin (Caliper, Waltham, MA, USA) in a PBS-solution was administered by intra-peritoneal injection with 150 mg luciferin/kg with an injection volume as 10 μL (D-luciferin PBS-solution)/g of body weight. In vivo luciferase imaging was captured by the IVIS Spectrum In Vivo Imaging System (Perkin Elmer, Waltham, MA, USA) 15 min after injection. The livers isolated from *pCAG EGFP* injected mice were perfused with 20 mL cold Hank’s buffer/mouse and assessed using a fluorescence stereoscope (SZX12 and DP-70, Olympus (Waltham, MA, USA)).

### 4.6. HTI of pCAG DA-Nrf2 and pCAG Nqo1 Vectors

For initial characterization, *pCAG* DA*-Nrf2*, *pCAG Nqo1* or their mock vectors, 25 μg of each vector + 25 μg of *pCAG Luciferase* + 5μg of *pRLTK-ΔARE* [47] were co-injected with the delivery solution. Livers were isolated 1 week later from each injected mouse and prepared for analyses. For modulating fatty livers in *Rosa^NIC/NIC^::AdiCre* mice, 5-week-old mice were injected with 25 μg of each *pCAG* vector. The plasma and livers were subsequently isolated 5 weeks after switching mice to the 5058 diet. Each sample was confirmed by PCR (Supplementary Figure S3). Primers and PCR conditions are shown in Supplementary Tables S3 and S4.

### 4.7. Mutagenesis for Construction of DA-Nrf2 cDNA

Wild type fragment of Neh2 domain was cloned by PCR using with 5-Nrf2-XN-ATG and 3-BamHI primers. This fragment and the rest Nrf2 coding region, BamHI-ApaI fragment from *pCMV Nrf2* [47] were linked into pBS KSII (Stratagene, San Diego, CA, USA) and termed pBS Nrf2. A 7K subdomain mutant of Neh2 was produced by PCR with 5′-KA-Nrf2 BglII and 3′-KA Nrf2 EcoRI primers cloned into BglII and EcoRI of pBS Nrf2 and termed *pBS 7K mut-Nrf2*. For *pBS DLG-7K-A Nrf2* mutant, a BsaBI-PstI DLG-7K Neh2 domain mutant fragment was created by PCR using both 5′-Nrf2 DLG-A SfcI and 3’-PstI-KA-Nhe2 primers and *pBS 7K mut-Nrf2* as template DNA. For *pBS 7K-ETGE-A Nrf2* mutant, a PstI-BamHI 7K-ETGE Neh2 domain mutant fragment was created by PCR using both 5′-PstI-LA-Nhe2 primer, 3′-Nrf2-ETGE-A-BamHI primer and pBS 7K mut-Nrf2 as template DNA. *pBS DLG-7K-A Nrf2* mutant and *pBS 7K-ETGE-A Nrf2* mutant was digested with XbaI, PstI and PstI, BamHI, respectively. Two mutated fragments were directly ligated between XbaI and BamHI of *pBSNrf2*. Point mutations were confirmed by sequencing analyses and the clone was termed *pBS* DA*-Nrf2*. The primers used in this construction are shown in Supplementary Table S5.

### 4.8. Construction of the Expression Vectors

*pRLTK-ΔARE*, *pCMV Keap1* and their mock vectors were constructed as described previously [48]. *pCAG EGFP* was provided by Dr. T. O’Connor (University of Tsukuba, Center for Tsukuba Advanced Research Alliance and Institute of Basic Medical Sciences). For construction of *pCAG Nqo1*, mouse Nqo1 cDNA was provided by Prof. P. Talalay [49]. NcoI, HindIII ORF fragment was subcloned into *pmKO1-S1*(MBL, Woburn, MA, USA). The modified *Nqo1* cDNA bearing the HindIII site blunted by T4 polymerase, cut out from this subclone with BamHI and then inserted into a *pTracer/EF vector* (Invitrogen, Waltham, MA, USA). Finally, the CAG promoter enhancer was placed into the region of the EF1a promoter. For the *pCAG Luciferase* construct the BamHI, SpeI fragment of CAG enhancer promoter region, which was isolated from the *pCAG Nrf2* vector, was cloned between BglII and NheI of the *pGL3 basic* vector (Promega, Madison, WI, USA). *pCAG Nrf2/*DA*-Nrf2*: Tracer/EF was prepared by dropping the BSD-cGFP expression unit into the same circular construct by SnaBI, PsiI treatment, then self-ligated. This new vector was used for the subcloning of mouse *Nrf2* or DA-*Nrf2* cDNA from the original pTracer/EF *Nrf2* or pTracer/EF DA-*Nrf2*, which was inserted into the wild-type mouse Nrf2 cDNA or DA-Nrf2 cDNA derived from each pBS clone described above. Consequently, each subclone’s EF1a promoter was switched with the CAG enhancer promoter region. The sequencing analyses of all vectors confirmed the recombination strategies.

### 4.9. Cell Culture, Transfection and Reporter Gene Assay

Mouse embryonic fibroblast (MEF) cells were established previously [47] and maintained in Iscove’s modified Dulbecco’s Medium (IMDM, Gibco, Waltham, MA, USA) containing 10% fetal bovine serum (FBS) and 100 μg/mL Primocin (InvivoGen, San Diego, CA, USA). Cells were incubated at 37 °C in a humidified atmosphere of 5% CO2. DNA mixtures included 2 μg *pNqo1-ARE Luc* [48], 10 μg each of *pCAG Nrf2*, *pCAG DA-Nrf2* or *pCAG Mock* with 2 μg each of *pCMV Keap1* or *pCMV Mock* [48] and 40 ng *pRLTK-ΔARE* as an internal control vector were transfected into wild-type MEF plated at 4 × 10^5^ cells/60 mm dish one day previous, by lipofection method [50] using Lipofectamine 2000 (Invitrogen, Waltham, MA, USA). Briefly, cells were then fed with Opti-MEM medium (Gibco, Waltham, MA, USA) at 35 °C in a humidified atmosphere of 5% CO_2_ incubator along with contact for 5 h with appropriate DNA mixture. Cells were then washed with PBS^-^ twice and refed with antibiotic-free regular culture medium. Transfectants were then incubated for 36 h until harvesting with 200 μL Passive lysis buffer (Promega, Madison, WI, USA). Luciferase activity was measured according to the manufacturer’s instructions (Dual luciferase assay [51] kit, Promega, Madison, WI, USA) and normalized to Renilla luciferase activity derived from *pRLTK-ΔARE*.

### 4.10. Immune Blotting Analyses

Proper-size cut tissues were homogenized in RIPA-I buffer [52], which contained a protease inhibitor cocktail (Roche, So., San Francisco, CA, USA). This solution was assayed for protein concentration by the Bio-Rad protein assay (Bio-Rad, Hercules, CA, USA) using bovine serum albumin (BSA) to generate a standard curve. A concentration of 10 mg/mL RIPA-I solution is ideal for immune blotting analyses. An equal volume of 2 × SDS sample buffer was added, and the samples were denatured by boiling for 5 min. Samples were applied onto SDS-PAGE [53] gels and transferred onto an Immobilon polyvinylidene difluoride (PVDF) membrane (Millipore, Burlington, MA, USA). The membranes were blocked with Tris-buffered saline with 0.05% Tween 20 and 5% skim milk (Difco, Tucker, GA, USA) and then treated with each primary antibody listed in Supplementary Table S6 in the Supplementary Materials. The preparative membranes were reacted with appropriate secondary antibodies conjugated to horseradish peroxidase (Invitrogen, Waltham, MA, USA). The immunocomplexes were visualized with ECL (PerkinElmer, Waltham, MA, USA). The detected band intensities were quantified by Image J and normalized by the nuclear Lamin B1 [54] band.

### 4.11. Histology

Mouse livers and epididymal adipose were isolated and fixed in 4% PFA, then embedded in paraffin, and sectioned. Sections were then deparaffinized using heat and xylene. Tissues were rehydrated using reducing concentrations of ethanol, then finally in water. Slides were stained with eosin and counterstained with hematoxylin (Thermo Fisher Scientific, Waltham, MA, USA). The sections were dehydrated using increasing concentrations of ethanol, cleared using xylene, and finally mounted using Paramount mounting medium (Thermo Fisher Scientific, Waltham, MA, USA). The images were viewed and recorded on a Nikon Eclipse E800 Core microscope (Nikon, Melville, NY, USA).

OCT blocks were prepared by embedding livers in OCT compound (Sakura Finetek USA, Torrance, CA, USA). Slides were fixed with 4% PFA at 4 °C for 10 min, then stained with oil Red O solution for 120 min. The liver sections were counterstained with hematoxylin (Carolina Biological Supply Company, Burlington, NC, USA).

### 4.12. Statistical Analysis

GraphPad Prism 9.5.1(528) was used for statistical analyses of data sets. Quantitative data are presented as mean ± SD. For the comparison of two groups, unpaired two-tailed Student’s *t* test was used; for more than two groups, one- or two-way ANOVA was used followed by Tukey’s or Dunnett’s test, as described in the relevant figure legends.

## Figures and Tables

**Table 1:** Genotypes of mice used in the research.

Genotype	Utility/Feature
*AdiCre*	Adipose tissue specific *Cre* expression: research control
*Rosa^NIC/NIC^*	Research control
*Rosa ^NIC/NIC^::AdiCre*	Lipodystrophy model mouse
*Kp1^A/A^:: Rosa ^NIC/NIC^::AdiCre*	Constitutive hypomorphic *Keap1* expression compound mouse
*Kp1^A/A^:: Rosa ^NIC/NIC^*	Control of *Kp1^A/A^::Rosa ^NIC/NIC^::AdiCre* mouse
*Kp1^B/B^:: Rosa ^NIC/NIC^::AdiCre*	Adipose tissue specific *Keap1* depleted compound mouse
*Kp1^B/B^:: Rosa ^NIC/NIC^*	Control of *Kp1^B/B^:: Rosa ^NIC/NIC^::AdiCre* mouse

## Data Availability

All data presented are contained within the manuscript/Supplementary Materials.

## Supplementary Materials

Supporting information can be downloaded at: https://www.mdpi.com/article/10.3390/ijms241713345/s1.
