# Supplementary material for: Forced Hepatic Expression of NRF2 or NQO1 Impedes Hepatocyte Lipid Accumulation in a Lipodystrophy Mouse Model
Source: Int J Mol Sci. 2023 Aug 28;24(17):13345. doi: 10.3390/ijms241713345 (PMC10487640; doi:10.3390/ijms241713345)

Table S1. Primers for mouse genotyping

Genotyping for Keap1$^{A/A}$ background mouse	
**Primers**	**Sequence (5' - 3')**
5-cko 4int1	GCACATCCTTCATCTCTCCGCACTGGGGAG
3-Kp1-4Ex	CCTCCGTGTCAACATTGGCGCGACTAG
R260-EGFP	GACTTGAAGAAGTCGTGCTGCTTCATGTG
Genotyping for Keap$^{B/B}$ background mouse	
Keap1-BF-F	CGAGGAAGCGTTTGCTTTAC
BF-R1	AGCCCCCTGCTGCATAGATAC
NeoI-3R	GAGTCACCGTAAGCCTGGTC
Keap1-4F	GAGTCCACAGTGTGTGGCC
Genotyping for Rosa$^{NIC/NIC}$ background mouse	
IMR0883	AAAGTCGCTCTGAGTTGTTAT
IMR8038	TAAGCCTGCCCAGAAGACTC
IMR8039	GAAAGACCGCGAAGAGTTTG
PGK-3740	GATGTGGAATGTGTGCGAGGCCAGAGGC
NICD-5477	GATTGTCGTCCATCAGAGCACCATCTGAGG
Genotyping for Cre background mouse	
Cre1	ACGTTCACCGGCATCAACGT
Cre2	CTGCATTACCGGTCGATGCA

Table S2. PCR programs for mouse genotyping

Keap1$^{A/A}$ background mouse			
**Step #**	**Temp °C**	**Time**	**Note**
1	95	1 min	-
2	95	30 sec	-
3	68.5	30 sec	-
4	72	30 sec	repeat steps 2-4 for 35 cycles
5	72	1 min	-
6	4	-	hold
Product; Flox A : ~350 bp, Disrupted : ~550 bp, Wt : ~250 bp			
Keap1$^{B/B}$ background mouse			
**Regular genotyping Primer: Keap1 BF-F and BF-R1**			
**Step #**	**Temp °C**	**Time**	**Note**
1	98	3 min	-

2	95	30 sec	-
3	68	30 sec	-
4	72	30 sec	repeat steps 2-4 for 35 cycles
5	4	-	hold
Product; Flox B : 445 bp, Wt : 267 bp			
**Excision confirmation Primer: Keap1-4F, BF-F and NeoI-3R**			
**Step #**	**Temp °C**	**Time**	**Note**
1	95	1 min	-
2	95	30 sec	-
3	68.5	30 sec	-
4	72	30 sec	repeat steps 2-4 for 35 cycles
5	72	1 min	-
6	4	-	hold
Product; Undisrupted : 383 bp, Disrupted : 288 bp, Wt : ~205 bp			
Rosa $^{NIC/NIC}$ **mouse** (https://www.jax.org/strain/008159) accessed 8/21/2023			
**Regular genotyping Primers: IMR0883, IMR8038 and IMR8039**			
**Step #**	**Temp °C**	**Time**	**Note**
1	94	3 min	-
2	94	30 sec	-
3	54	1 min	-
4	72	1 min	repeat steps 2-4 for 35 cycles
5	72	2 min	-
6	10	-	hold
Product; Tg : 320 bp, Wt : 235 bp			
Rosa ^{NIC} **active allele by Cre Primers; IMR0883, NICD-5477 and PGK-3740**			
**Step #**	**Temp °C**	**Time**	**Note**
1	95	1 min	-
2	95	30 sec	-
3	65.4	30 sec	-
4	72	45 sec	repeat steps 2-4 for 35 cycles
5	72	2 min	-
6	4	-	hold
Product; Cre-Active : 650 bp, Cre-Inactive : 550 bp			
Cre transgenic allele			
**Step #**	**Temp °C**	**Time**	**Note**
1	94	1 min	-
2	94	30 sec	-

3	60	30 sec	-
4	72	30 sec	repeat steps 2-4 for 35 cycles
5	4	-	hold
Product; *Cre*-Tg : 355 bp			

Table S3. Primers for transgene confirmation

Primers	Sequence (5' - 3')
CAGGs	CTCTAGAGCCTCTGCTAACC
PB-6679080a1-R	AGGCGTCCTTCCTTATATGCTA
pCAG-F	GCAACGTGCTGGTTATTGTG
PB-6754832a1-R	GTTGAAACTGAGCGAAAAAGGC

Table S4. PCR program for HTI-transgene confirmation

Primer for DA-*Nrf2*: pCAG-F and PB-6754832a1-R

Primer for *Nqo1*: CAGGs and PB-6679080a1-R

Step #	Temp °C	Time	Note
1	94	1 min	-
2	95	30 sec	-
3	65.7	30 sec	-
4	72	20 sec	repeat steps 2-4 for 35 cycles
5	4	-	hold

Product; DA-*Nrf2* : 294 bp, *Nqo1*: 401bp

Table S5. Primers used in mutagenesis

Primer	Sequence (5' - 3')
5-Nrf2-XN-ATG	CTAGTCTAGACATATGATGGACTTGGAGTTGCCACCGCC
5-Nrf2 DLG-A SfcI	AGGACTACAGTCCCAGCAGGACATGGATTTGATTGACATCGCATGGAGGGCAGCAAT AGCAGCTGCAGTAAGTCGAGAAGTGTTTGACTTTAGTCAG
5-KA-Nrf2 BglII	ATAGATCTTGGAGTAAGTCGAGAAGTGTTTGACTTTAGTCAGCGACAGGCAGACTATG AGCTGGAAGCACAGGCAGCACTCGAAGCGGAAAGACAAGAGC
5'-PstI-KA-Nhe2	GAGCAACTGCAGGCGGAACAGGAGGCGG
3'-PstI-KA-Nhe2	TTCCGCCTGCAGTTGCTCTTGTCTTTCC
3-BamHI	CCTGGGAGTAGCTGGCGGATCCACTG
3-KA Nrf2 EcoRI	AGGAATTCTCCTGTTTCTTCATCCAGTTGAAACTGAGCGAAAAAGGCCGCCTCCTGTTC CGCCTGGAGTTGCTCTTGTCTTTCCGCTTCGAGTGCTGCCT

Table S6. Antibodies used in experiments

Target Protein	Provider	Dilution
Nrf2	Invitrogen PA5-27882	2,000
LaminB1	Proteintech 12987-1-AP	5,000
Nqo1	Abcam ab2346	IB, IHC: x 500
Gclc	Proteintech 12601-1-AP	2,000
GstA1-5	Invitrogen PA5-79335	2,000
Acc1	Proteintech 21923-1-AP	1,000
Fasn	Proteintech 10624-2-AP	1,000
Keap1	Original	3,000
Luciferase	Novus Biological NB100-1677	1,000
Rabbit anti-Goat IgG (H+L)-HRP	Invitrogen 31402	10,000
Goat Anti-Rabbit IgG (H + L)-HRP	BIO RAD 1706515	3,000
Horse anti-Goat IgG (H+L), Biotinylated	Vector Laboratories BA-9500	x 200

Supplemental Figure S1. ***Keap1*** **and flox mutant gene structure and primer positions for genotyping.** (A) *Kp1*$^{A/A}$ flox allele. (B) *Kp1*$^{B/B}$ flox allele. Grey and blue boxes indicate non-coding and coding region of each exon of *Keap1*gene. The triangles show position of flanking lox sequences in mutant mice.

Supplemental Figure S2. Representative results of confirmative genotyping. The positive controls were from tail DNA isolated from each heterozygote of *Kp1*$^{A/+}$, *Kp1*$^{B/+}$, *Rosa*$^{NIC/+}$ and *Rosa*$^{NIC/+}$*::AdiCre* mice for *Keap1 A flox* (top), *Keap1 B flox* (second), *Rosa* (third), and *Adipoq Cre* (bottom) genotyping, respectively.

Supplemental Figure S3. Confirmation of transgene by HTI. The primer set positions for *Nqo1* (CAGGs, PB-6679080a-1-R) and *DA-NRF2* expression vectors (pCAG-F, PB-6754832a1-R) are depicted in (A). The representative PCR results are shown in (B). White, steel grey and mercury boxes show the representative result of transgene detection from *pCAG Mock*, *pCAG Nqo1* and *pCAG DA-Nrf2* HTI mice (N=4), respectively. 1μL of template DNA was utilized as control which includes 10ng of each plasmid DNA mixed with wild-type tail genomic DNA treated as per usual genotyping.

Supplemental Figure S4. Immunohistochemical analysis of NQO1 expression in the HTI-liver of ***Rosa***$^{NIC/NIC}$***::AdiCre*** **mice.** 5-week old male *Rosa*$^{NIC/NIC}$*::AdiCre* mice were hydrodynamically injected with *pCAG Mock* (A) or *pCAG Nqo1* (B) through the tail vein. Five weeks following HTI and feeding with HFD, livers were isolated from the mice and its sections were prepared and analyzed with anti-NQO1 antibody immunohistochemically. NQO1 derived from p*CAG Nqo1* HTI liver was stained strongly by the precipitated DAB reaction product. Scale bar: 100 μm.

Figure S1

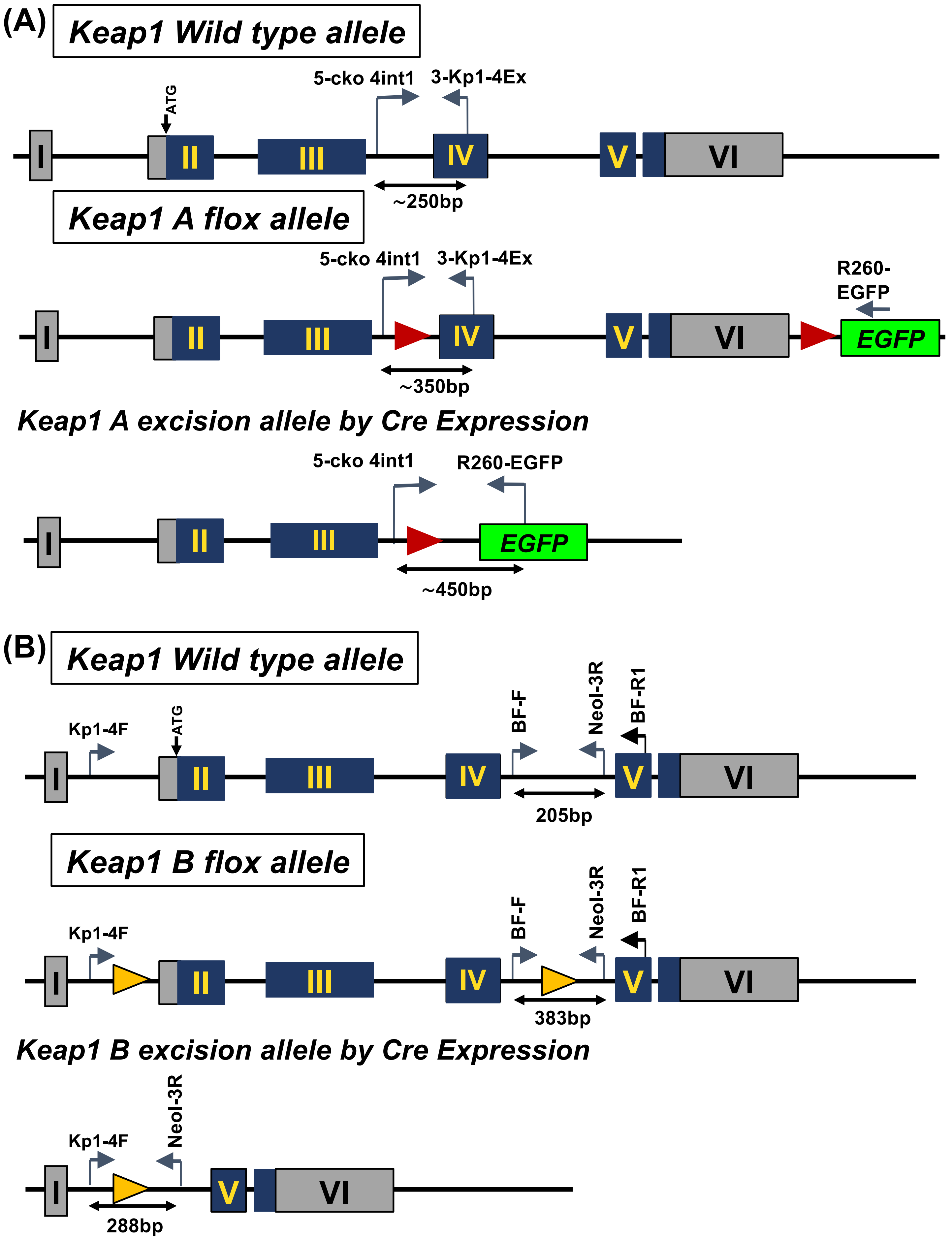
(A)
Keap1 Wild type allele
ATG
5-cko 4int1
3-Kp1-4Ex
I
II
III
IV
V
VI
~250bp
Keap1 A flox allele
5-cko 4int1
3-Kp1-4Ex
R260-EGFP
EGFP
~350bp
Keap1 A excision allele by Cre Expression
5-cko 4int1
R260-EGFP
EGFP
~450bp
(B)
Keap1 Wild type allele
Kp1-4F
ATG
BF-F
Neol-3R
BF-R1
205bp
Keap1 B flox allele
Kp1-4F
BF-F
Neol-3R
BF-R1
383bp
Keap1 B excision allele by Cre Expression
Kp1-4F
Neol-3R
288bp

Figure S2

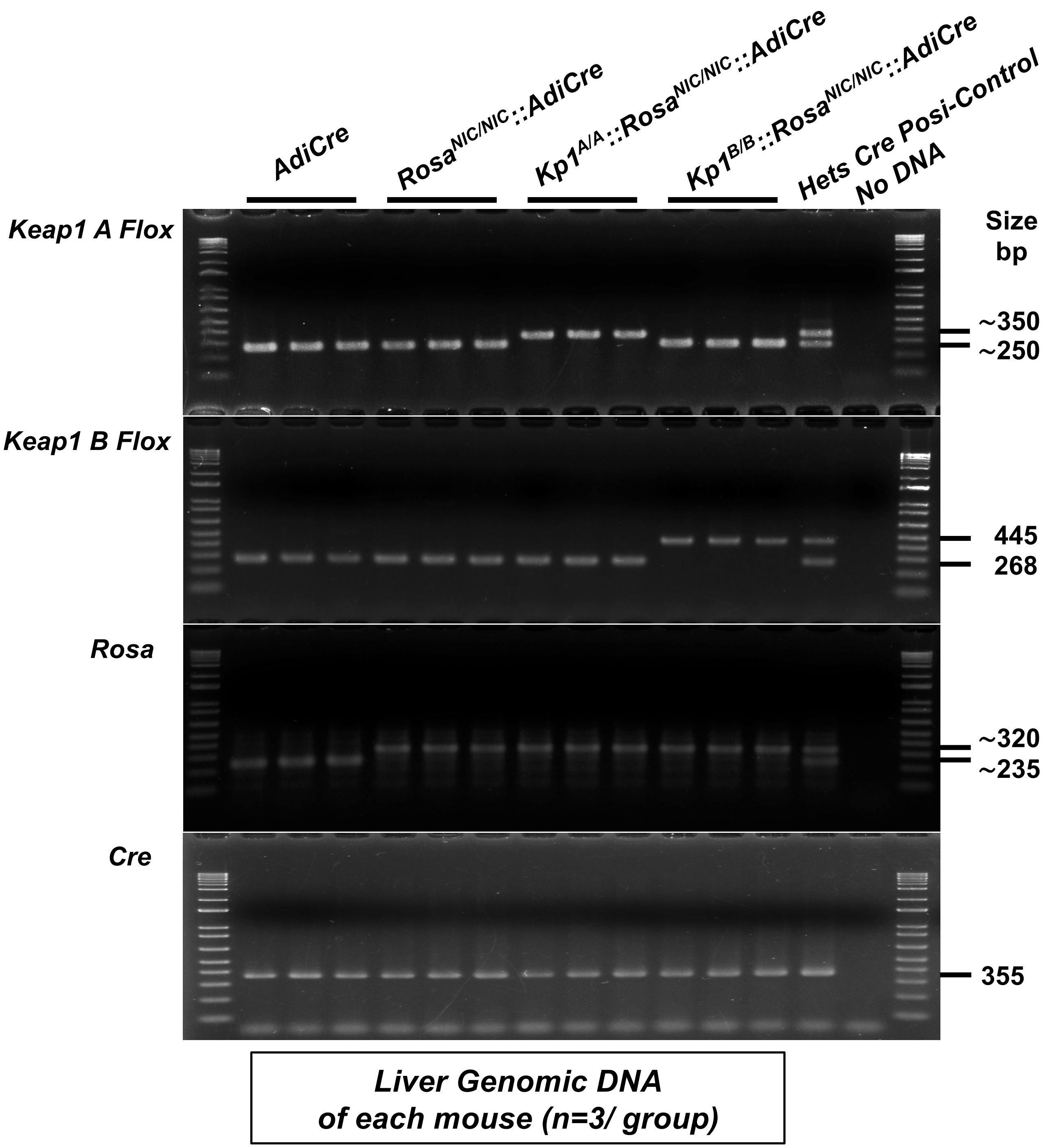
AdiCre
Rosa^NIC/NIC::AdiCre
Kp1^A/A::Rosa^NIC/NIC::AdiCre
Kp1^B/B::Rosa^NIC/NIC::AdiCre
Hets Cre Posi-Control
No DNA
Keap1 A Flox
Keap1 B Flox
Rosa
Cre
Size bp
~350
~250
445
268
~320
~235
355
Liver Genomic DNA
of each mouse (n=3/ group)

Figure S3

(A)

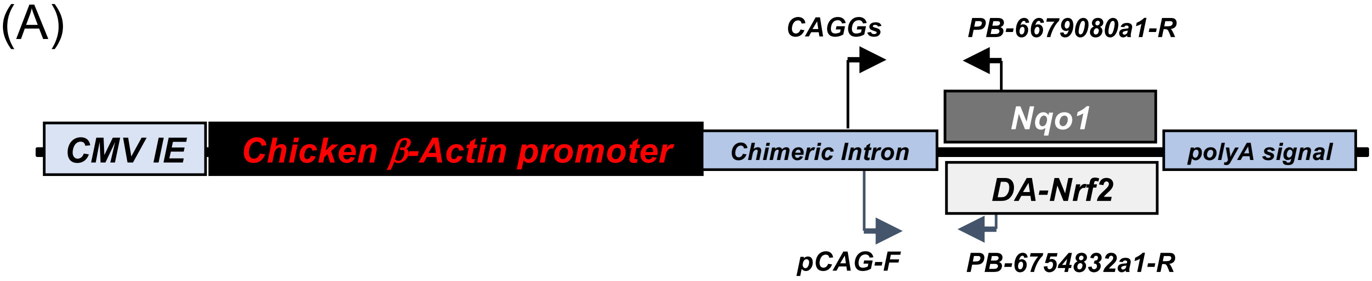
CAGGs
PB-6679080a1-R
Nqo1
CMV IE
Chicken β-Actin promoter
Chimeric Intron
polyA signal
DA-Nrf2
pCAG-F
PB-6754832a1-R

(B)

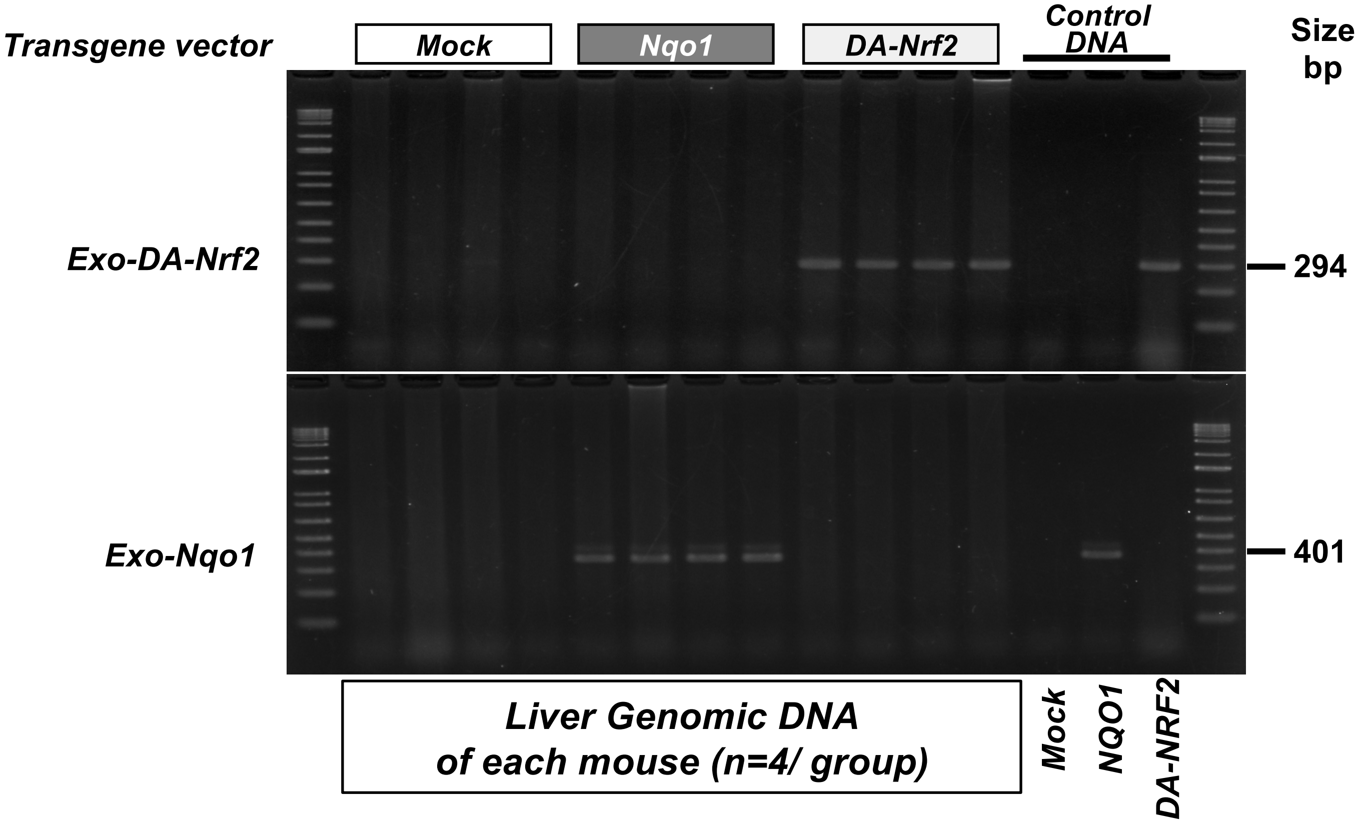
Transgene vector
Mock
Nqo1
DA-Nrf2
Control DNA
Size bp
Exo-DA-Nrf2
294
Exo-Nqo1
401
Liver Genomic DNA
of each mouse (n=4/ group)
Mock
NQO1
DA-NRF2

Figure S4

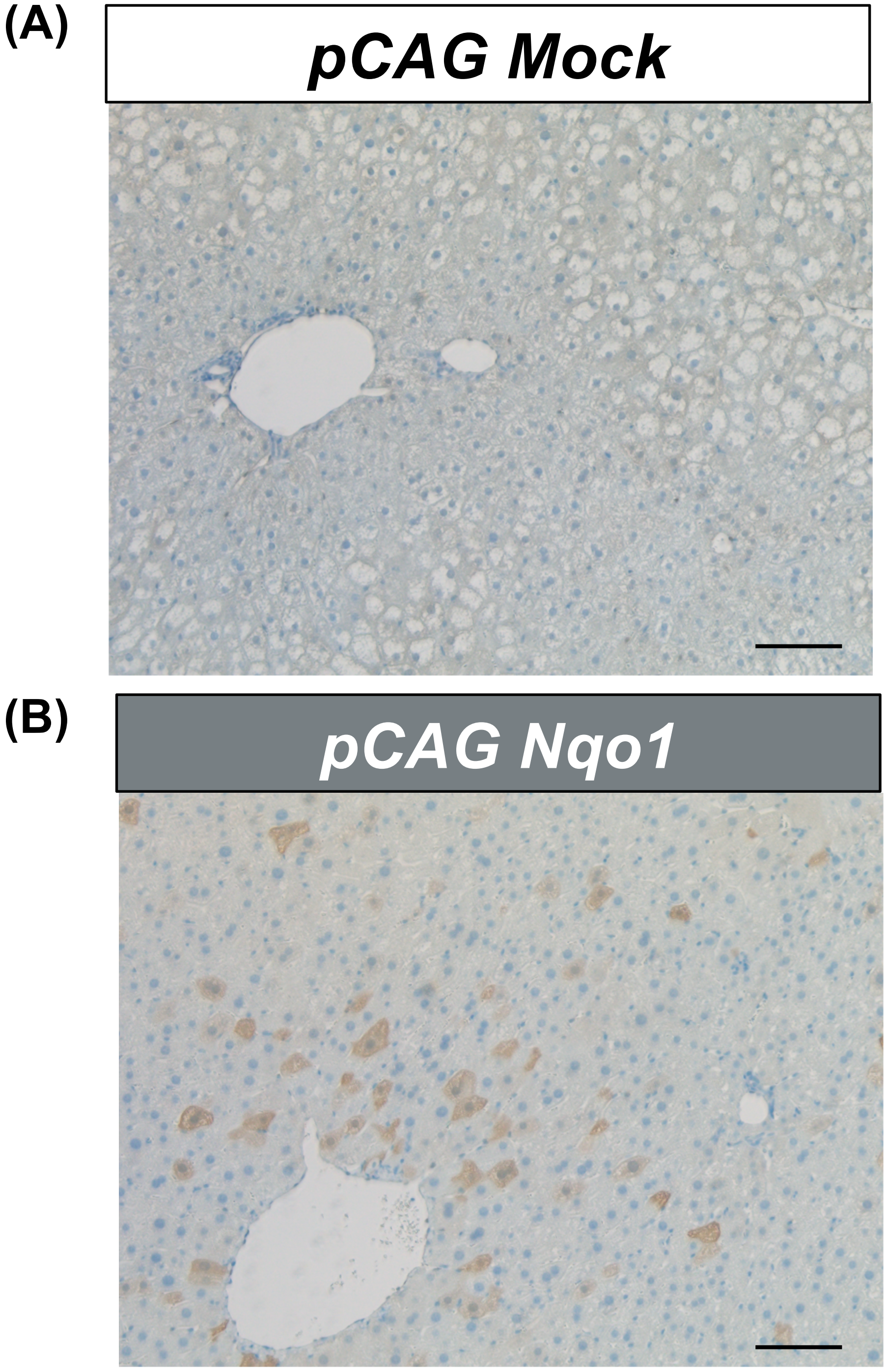

Supplement: Supplementary file 1 [file ijms-24-13345-s001.zip › ijms-2522905-supplementary.pdf]
